# Refining the *Schistosoma haematobium* recombinase polymerase amplification (Sh-RPA) assay: moving towards point-of-care use in endemic settings

**DOI:** 10.1186/s13071-024-06380-9

**Published:** 2024-07-28

**Authors:** Owain Donnelly, Silvia Mesquita, John Archer, Said M. Ali, Zikmund Bartonicek, Elena B. Lugli, Bonnie L. Webster

**Affiliations:** 1 Wolfson Wellcome Biomedical Laboratories, Department of Science, Natural History Museum, Cromwell Road, London, SW7 5BD UK; 2 Malaria Biochemistry Laboratory, The Francis Crick Institute, 1 Midland Road, London, NW1 1AT UK; 3 René Rachou Institute, Oswaldo Cruz Foundation, Rio de Janeiro, Brazil; 4 Department of Parasitology, Liverpool School of Tropical Medicine, Liverpool, L3 5QA UK; 5 Public Health Laboratory-Ivo de Carneri, P.O. Box 122, Chake-Chake, Pemba United Republic of Tanzania; 6 Department of Genetics, Evolution and Environment, University College London, London, UK

**Keywords:** Diagnostic, Molecular, Isothermal, Point-of-care, Schistosomiasis, *Schistosoma haematobium*, Urogenital schistosomiasis, Recombinase polymerase amplification

## Abstract

**Background:**

Urogenital schistosomiasis is caused by the parasitic trematode *Schistosoma haematobium*. Sensitive and specific point-of-care diagnostics are needed for elimination of this disease. Recombinase polymerase amplification (RPA) assays meet these criteria, and an assay to diagnose *S. haematobium* has been developed (Sh-RPA). However, false-positive results can occur, and optimisation of reaction conditions to mitigate these is needed. Ease of use and compatibility of DNA extraction methods must also be considered.

**Methods:**

Using synthetic DNA, *S. haematobium* genomic DNA (gDNA), and urine samples from clinical cases, Sh-RPA reactions incorporating different betaine concentrations (0 M, 1 M, 2.5 M, 12.5 M) and the sample-to-water ratios were tested to determine effects on assay specificity and sensitivity. In addition, five commercial DNA extraction kits suitable for use in resource-limited settings were used to obtain gDNA from single *S. haematobium* eggs and evaluated in terms of DNA quality, quantity, and compatibility with the Sh-RPA assay. All samples were also evaluated by quantitative polymerase chain reaction (qPCR) to confirm DNA acquisition.

**Results:**

The analytical sensitivity of the Sh-RPA with all betaine concentrations was ≥ 10 copies of the synthetic *Dra1* standard and 0.1 pg of *S. haematobium* gDNA. The addition of betaine improved Sh-RPA assay specificity in all reaction conditions, and the addition of 2.5 M of betaine together with the maximal possible sample volume of 12.7 µl proved to be the optimum reaction conditions. DNA was successfully isolated from a single *S. haematobium* egg using all five commercial DNA extraction kits, but the Sh-RPA performance of these kits varied, with one proving to be incompatible with RPA reactions.

**Conclusions:**

The addition of 2.5 M of betaine to Sh-RPA reactions improved reaction specificity whilst having no detrimental effect on sensitivity. This increases the robustness of the assay, advancing the feasibility of using the Sh-RPA assay in resource-limited settings. The testing of commercial extraction kits proved that crude, rapid, and simple methods are sufficient for obtaining DNA from single *S. haematobium* eggs, and that these extracts can be used with Sh-RPA in most cases. However, the observed incompatibility of specific kits with Sh-RPA highlights the need for each stage of a molecular diagnostic platform to be robustly tested prior to implementation.

**Graphical Abstract:**

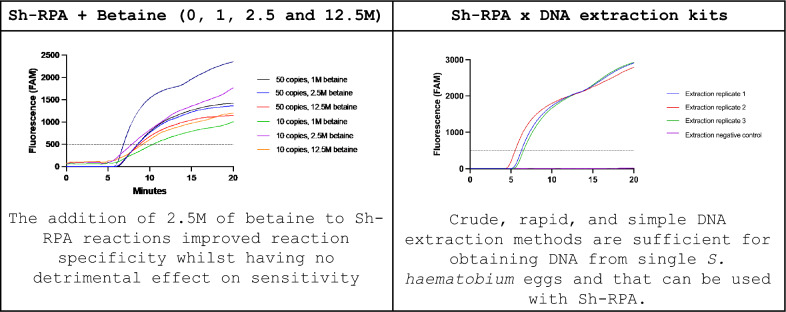

**Supplementary Information:**

The online version contains supplementary material available at 10.1186/s13071-024-06380-9.

## Background

Schistosomiasis is a debilitating neglected tropical disease (NTD) caused by infection with parasitic trematodes of the genus *Schistosoma*, affecting over 230 million people in 78 countries [1]. The total disease burden caused by schistosomiasis is estimated to be over 1.6 million disability-adjusted life years across South America, sub-Saharan Africa, and Asia [2, 3]. However, over 85% of all cases occur in sub-Saharan Africa, where *Schistosoma haematobium*, the causative species of urogenital schistosomiasis, is responsible for approximately two-thirds of cases [4, 5].

*Schistosoma haematobium* is unique with regard to its anatomical site of infection in humans, with adult worms inhabiting the venous plexus of the bladder and females excreting eggs which pass through the bladder tissue into the urine of infected individuals. Excreting this urine into fresh water propagates onward transmission via aquatic snail intermediate hosts of the genus *Bulinus*. However, the majority of eggs become inadvertently sequestered in tissues rather than passed into the urinary tract, resulting in tissue inflammation and the pathological manifestations of urogenital schistosomiasis [6]. These can include haematuria, bladder calcification, hydronephrosis, and an increased risk of bladder cancer [1, 7]. In addition, female and male genital schistosomiasis (FGS/MGS), caused by egg deposition in pelvic organs, can result in sexual health and reproductive problems in women, erectile and prostatic problems in men, and increased risk of HIV acquisition/transmission, all of which can lead to stigmatisation [8–13].

Public health interventions to combat schistosomiasis have focused primarily on reducing morbidity via mass drug administration (MDA) of the oral anthelminthic drug praziquantel [14, 15]. This approach has been very successful in many endemic countries, with the World Health Organization (WHO) now proposing to move beyond morbidity reduction strategies towards disease elimination as a public health problem, defined as reducing the prevalence of high-intensity infections to fewer than 1% of the population [16]. As such, reliable and robust diagnostic tools capable of detecting low levels of infection in resource-limited settings are needed to achieve, monitor, and sustain disease elimination [17, 18]. Moreover, these diagnostic tools need to be accessible, acceptable, and pragmatically usable in the settings in which they are needed.

Current methods for diagnosing *S. haematobium* infection, however, lack sensitivity and/or specificity [19]. For example, the routine diagnostic method of urine-egg microscopy lacks sensitivity, particularly when attempting to identify low-intensity infections [20–22]. Alternative diagnostic assays are available, such as antigen tests (point-of-care circulating cathodic antigen [POC-CCA], circulating anodic antigen [CAA]), lateral-flow dipsticks that detect haematuria, and serology tests used to detect *S. haematobium* antibodies; however, these lack either high sensitivity or high specificity, or both [23–25]. A laboratory-based CAA test (up-converting-phosphor lateral-flow [UCP-LF] CAA) shows high levels of sensitivity and specificity but is not currently available as a point-of-care test [26].

Molecular diagnostic platforms are highly sensitive and specific. Polymerase chain reaction (PCR)-based diagnostics, for example, can be used as a reference test to reliably diagnose *S. haematobium* infection by amplifying and detecting species-specific DNA within urine samples [27, 28]. However, PCR is sensitive to inhibitors, and requires careful sample preparation as well as specialised laboratory infrastructure, expensive equipment, and skilled personnel, limiting its use in resource-poor settings such as those endemic for schistosomiasis [19].

Portable and isothermal molecular diagnostics, such as loop-mediated isothermal amplification (LAMP) and recombinase polymerase/assisted amplification (RPA/RAA), overcome some of these limitations, presenting opportunities for point-of-care use. A real-time *S. haematobium* RPA assay targeting the genomic *Dra1* tandem repeat region (the RT-ShDra1-RPA) has been used to reliably detect infection within individuals expelling extremely low numbers of eggs (< 10 eggs/10 ml urine) [29]. In addition, this assay was also adapted to diagnose FGS by detecting low levels of *S. haematobium* DNA within cervicovaginal lavage and less-invasive vaginal self-swab samples (the Sh-RPA) [30]. Although this assay has proven to be sensitive and specific, further refinements are needed to make it more robust and feasible for use in low-resource settings by personnel with limited training.

For example, due to the relatively low temperatures (usually 37–42 °C) used for RPA reactions compared to PCR and LAMP, false-positive results can occur due to secondary structure formation [31–33]. Understanding and minimising false-positive results is therefore essential before upscaling routine use of RPA for diagnostic purposes at the point of care. The addition of betaine to PCR, LAMP, and RPA reactions has been used to successfully reduce false positives [32–34], as well as to facilitate DNA amplification when the guanine/cytosine (GC) content is high [35] and lower the melting temperature of DNA in reactions, thus increasing reaction efficiency [36]. Another factor limiting any molecular assay’s use at the point of care is sample preparation and the simplicity of reaction set-up. Due to the enzymes used in LAMP and RPA reactions, sample preparation methods which generate more ‘crude’ template DNA can be used, as these assays are more tolerant of reaction inhibitors within the sample compared to PCR-based approaches [37, 38].

Here, we investigate the impact of using betaine in Sh-RPA reactions and its effect on assay sensitivity and specificity. We also explore different sample preparation methodologies, as well as how template DNA volume within the reaction affects assay sensitivity, with the aim of further optimising the Sh-RPA for use in low-resource settings.

## Methods

### Study samples and DNA templates/standards

#### Synthetic *S. haematobium* (Sh) Dra1 DNA standard

A synthetic DNA standard incorporating the 121-base-pair (bp) *Dra1* repeat region (GenBank accession number: DQ157698.1), together with 3′ and 5′ flanking regions (Supplementary Table S1), was commercially synthesised by GeneArt (Invitrogen, USA) and used as a synthetic DNA standard [29]. The synthetic DNA stock solution was diluted to a working concentration of 1 × 10^8^ copies/µl using double-distilled water (ddH_2_O). Ten-fold serial dilutions were then performed to produce working solutions ranging between 1 × 10^8^ and 1 × 10^1^ copies/µl.

#### Single *S. haematobium* (Sh) eggs

*Schistosoma haematobium* (Egyptian strain) eggs were provided frozen by the Schistosomiasis Resource Center (Biomedical Research Institute, USA, https://www.afbr-bri.org/schistosomiasis/) [39, 40]. The eggs were defrosted at room temperature, diluted in ddH_2_O, and single eggs were isolated using a micropipette under a dissecting microscope. Single eggs were used in the different experiments as described below. Unless otherwise specified, genomic DNA (gDNA) was extracted from individual eggs using the SpeedXtract Nucleic Acid Kit (Qiagen) as described previously [29].

#### Genomic *S. haematobium *adult worm DNA (Sh gDNA)

Genomic DNA isolated from adult *S. haematobium* worms originating from Nigeria was provided by the Schistosomiasis Collection at the Natural History Museum (SCAN) [41]. Genomic DNA was isolated using the DNeasy Blood & Tissue Kit (Qiagen) according to manufacturer’s instructions and quantified using a NanoDrop 3300 Fluorospectrometer (Thermo Fisher Scientific, USA). The stock gDNA solution was diluted to a concentration of 1 ng/µl using ddH_2_O, which was then also serially diluted to produce working solutions ranging between 1 pg/µl and 1 fg/µl.

#### Urine samples: gDNA extracted from the urine of *S. haematobium*-infected individuals in Zanzibar

DNA isolated from six urine samples used for initial Sh-RPA pilot testing as described previously [29, 42] were selected and used for further assay optimisation. DNA was previously isolated from these urine samples using the SpeedXtract Nucleic Acid Kit (Qiagen) as described previously [29]. All six samples had a mean urine-egg count of 1–10 eggs per 10 ml urine, as measured by urine-egg microscopy, and were therefore deemed ‘very low egg count’ (Table 1). Urine samples were originally collected as part of the Zanzibar Elimination of Schistosomiasis Transmission (ZEST) project, which ran from 2011 to 2017 [43].

**Table 1:** Details of the six urine samples with their corresponding egg counts, collected from *S. haematobium*-positive individuals, that were processed and analysed by the Sh-RPA as described previously [29, 30, 42]

	Eggs per 10 ml of urine		
Sample ID	Count 1	Count 2	Mean egg count
U3	8	10	9
U16	3	0	1.5
U17	1	0	0.5
U18	2	0	1
U19	3	9	6
U20	1	0	0.5

### Sh-RPA reactions

#### Sh-RPA reaction set-up

Sh-RPA reactions were performed using the TwistAmp exo kit (TwistDx, Cambridge, UK), and all reactions were carried out using the AmpliFire isothermal fluorometer (Douglas Scientific, USA) as described previously [29, 30]. Reactions contained 2.1 µl of each Sh-RPA primer, 0.6 µl of the Sh-RPA fluorophore-labelled probe (primer and probe were used at the concentration of 10 µM and their sequences are described in [42] and replicated in Supplementary Table S1), 29.5 µl rehydration buffer, 2.5 µl (280 nM) magnesium acetate (MgAc), and the lyophilised RPA pellet, which contains all the enzymes and other components necessary for the RPA reaction. Depending on the experiment being conducted, different volumes of betaine (5 M, Sigma-Aldrich, USA), DNA, and ddH_2_O were added, so that the total reaction volume was always 50 µl.

Reactions were carried out by preparing a master mix containing the primers, probe, rehydration buffer, betaine, and ddH_2_O. The master mix was then added to each lyophilised RPA pellet, and 2.5 µl of MgAc was pipetted into tube lids. The lids were then carefully closed so that the MgAc was not introduced into the reaction mix prematurely, as this would initiate the reaction. After this, the template DNA or control was added to each tube, with the tube lid being opened and closed each time to limit contamination. Next, reaction tubes were briefly centrifuged then inverted by hand (~5 times) and promptly centrifuged again before being placed in an isothermal fluorometer (AmpliFire, Douglas Scientific, USA). Reactions were run at 42 °C for 20 min, with a manual mix and brief centrifuge of the reactions at 4 min into the run time. The AmpliFire recorded the fluorescence of each reaction over time.

Samples were considered positive if an increase from baseline of ≥ 500 relative fluorescence units (RFU) was recorded by the isothermal fluorometer. Each set of reactions contained positive (*Dra1* standard 5 × 10^1^ or 1 pg of adult *S. haematobium* worm gDNA) and negative (no-template ddH_2_O) controls. The different experimental reaction compositions tested are shown in Table 2 and described below.

**Table 2:**
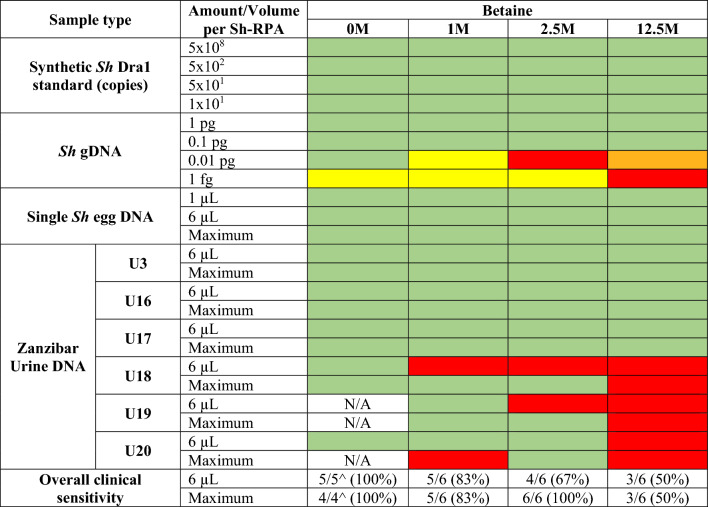
Data from the Sh-RPA reactions performed with different sample types and amounts, together with different amounts of betaine included in the reaction

Each cell in the table represents the replicates of each Sh-RPA condition tested. * = Sh-RPA was positive but the run was compromised by a positive result in the negative control; N/A = not enough sample stored to complete this reaction. Maximum = the maximum sample volume that could be added to the Sh-RPA (replacing all ddH_2_O in the reaction mix). ^ = Denominator affected by limited availability of clinical samples for Sh-RPA. This equates to 13 µl of sample in reactions containing 1 M of betaine, 12.7 µl of sample in reactions containing 2.5 M of betaine, and 10.7 µl of sample in reactions containing 12.5 M of betaine, respectively. Coloured cells represent number of positive replicates from the total of three replicates; green = 3/3, yellow = 2/3, orange = 1/3, red = 0/3

#### Effects of betaine on the analytical sensitivity and specificity of the Sh-RPA assay

To assess the effect of betaine on the analytical sensitivity and specificity of the Sh-RPA assay, reactions were performed containing different amounts of betaine and different concentrations of the synthetic *Sh Dra1* DNA standard or *Sh* gDNA, as shown in Table 2. Initially, the analytical limit of detection (LOD) of Sh-RPA reactions was tested using both synthetic *Sh Dra1* DNA standards (5 × 10^8^, 5 × 10^2^, 5 × 10^1^, and 1 × 10^1^) and ten-fold-diluted concentrations of *Sh* gDNA (from 1 pg to 1 fg), with varying amounts of betaine (0 M, 1 M, 2.5 M, or 12.5 M) as detailed in Table 2. Negative control Sh-RPA reactions, containing ddH_2_O in place of template DNA, were carried out with each of the betaine amounts (1 M, 2.5 M, or 12.5 M). Seven replicates of each negative control were performed.

#### Effects of sample volume and betaine concentration on Sh-RPA performance

Sh-RPA reactions were tested using different volumes of crude DNA isolated from single *S. haematobium* eggs to determine any effects on reaction sensitivity and inhibition. Sh-RPA reactions were performed as described above but with varying volumes of sample and betaine.

Triplicate reactions were performed using 1, 6, 10.7, or 12.7 µl of gDNA extracted from single *S. haematobium* eggs, with each reaction containing either 0 M, 1 M, 2.5 M, or 12.5 M of betaine as shown in Table 2. This was then repeated for the clinical urine samples (described in Table 1) with Sh-RPA reactions performed using combinations of 1, 6, 10.7, or 12.7 µl of gDNA from each sample and 0 M, 1 M, 2.5 M, or 12.5 M of betaine (Table 2). For all reactions, the volume of ddH_2_O was varied within each reaction to maintain a 50 µl total reaction volume. However, some reactions contained no ddH_2_O, as the maximum sample volume (10.7 or 12.7 µl) was added.

#### Assessment of different DNA extraction methods for use with Sh-RPA

Five commercial DNA extraction kits characterised by ease, speed, and suitability for use in low-resource settings were tested: Extracta DNA Prep (Quantabio), DNeasy Blood & Tissue Kit (Qiagen), Genesig Easy DNA/RNA Kit (Primerdesign), SpeedXtract Nucleic Acid Kit (Qiagen), and SwiftX DNA (Xpedite Diagnostics). Aliquots of ddH_2_O (100 µl) were spiked with single *S. haematobium* eggs and then processed using the different DNA extraction kits with some modifications to the manufacturer’s protocols. Extractions were performed in triplicate, and a negative no-template control for each extraction kit was performed using only 100 µl ddH_2_O and no egg.

Where heating was needed for the sample lysis step, two sets of samples were processed for each: one set at the recommended temperature (95 °C for the Extracta DNA Prep [Quantabio] kit and SwiftX DNA [Xpedite Diagnostics], and 56 °C for the DNeasy Blood & Tissue Kit [Qiagen]), and the other set at room temperature (± 19 °C), with the latter removing the need for a heating block. In addition, SwiftX DNA extractions were performed using a smaller volume of reagents than that recommended by the manufacturer (7.5 µl of magnetic beads and 50 µl of lysis buffer DL), with the aim of reducing cost per extraction.

To assess the performance of the different DNA extraction methods on the single *S. haematobium* eggs, and to rule out false-negative Sh-RPA outcomes due to unsuccessful DNA extraction, each DNA isolate was tested using a standard diagnostic quantitative PCR (qPCR) targeting a genus-specific internal transcribed spacer (ITS) region [44, 45]. Reactions were performed in a final volume of 20 µl containing 1× Luna Universal Probe qPCR MasterMix (New England Biolabs), 200 nM of each primer, 50 nM of the probe and 1 µl of extracted DNA. All reactions were carried out in triplicate on a StepOnePlus real-time PCR instrument (Applied Biosystems, USA) using the following cycling conditions: 10 min initial denaturation and 50 cycles of 95 °C denaturation for 15 s followed by combined 60 °C annealing/extension for 60 s. Positive controls (1 µl of *S. haematobium* egg gDNA) and no-template negative controls (ddH_2_O) were included with each qPCR reaction. To quantify DNA, a standard curve with ten-fold serial dilutions ranging from 100 pg to 10 fg of *S. haematobium* worm gDNA was generated and used in each set of qPCR reactions for quantitative analysis. DNA extraction was considered successful if a cycle threshold (Ct) value of < 38 was given.

Standard Sh-RPA reactions were then performed containing 2.5 M of betaine and the maximum sample volume (12.7 µl), obviating the need to add ddH_2_O. Additionally, whole single *S. haematobium* eggs were suspended in 12.7 µl of ddH_2_O and then added directly to an Sh-RPA reaction without any processing. Details and diagnostic outcome for each DNA extraction method used are described in Table 3.

**Table 3:** Comparison of DNA extraction kits tested with the Sh-RPA assay

Extraction kit	Sample lysis temperature (°C)	Estimated time to extract 8 samples (min)	Cost per extraction (USD)^b^	Heat block required	Magnetic bead method^c^	Mean qPCR Ct value (± SD)	Sh-RPA diagnostic outcome	Sensitivity (%)
Extracta DNA Prep (Quantabio)	95^a^	15	$0.34	Y	N	24.6 (± 1.9)	** + **	3/3 (100%)
	Room temperature (± 19)			N		24.0 (± 0.6)	** + **	3/3 (100%)
DNeasy Blood & Tissue Kit (Qiagen)	56^a^	30	$4.25	Y	Y	24.7 (± 1.4)	** + **	2/3 (67%)
	Room temperature (± 19)			N		24.9 (± 1.7)	** + **	2/3 (67%)
Genesig Easy DNA/RNA Kit (Genesig)	Room temperature (± 19)^a^	30	$6.34	N	Y	25.5 (± 1.2)	** − **	0/3 (0%)
SpeedXtract Nucleic Acid Kit (Qiagen)^d^	95^a^	15	N/A^d^	Y	Y	26.5 (± 0.7)	** + **	1/1 (100%)^f^
SwiftX DNA (Xpedite Diagnostics)^e^	95^a^	15	$3.20	Y	Y	32.9 (± 2.8)	+	3/3 (100%)
	Room temperature (± 19)			N		34.7 (± 2.1)	** + **	3/3 (100%)

The SpeedXtract Nucleic Acid Kit (Qiagen) protocols can be modified to remove the need for heating, although this could not be tested at the time of this study

^a^Manufacturer-recommended temperature for sample lysis step

^b^Costs estimated as of November 2022

^c^Magnetic bead-based methods are portable and enable capture of the DNA, which could increase sensitivity

^d^The SpeedXtract Nucleic Acid Kit (Qiagen) has been discontinued, and an equivalent kit is now manufactured by Xpedite Diagnostics (https://www.xpedite-dx.com) and is called the SwiftX DNA Kit

^e^Used an extraction protocol modified from that recommended by the manufacturer, using a smaller volume of magnetic beads and extraction buffer

^f^Due to discontinuation of the SpeedXtract kit, only one replicate of Sh-RPA was undertaken

## Results

### Effects of betaine on Sh-RPA sensitivity and specificity

No difference was observed in the performance of the Sh-RPA assay for the detection of the synthetic *Sh Dra1* DNA standards, with and without the addition of 1 M, 2.5 M, and 12.5 M of betaine to Sh-RPA reactions (Table 2). There was an observable difference in time to onset of amplification for the different concentrations of synthetic *Sh Dra1* DNA standard tested, with earlier amplification onset when using greater concentrations of synthetic DNA (Supplementary Figure S1). In addition, the Sh-RPA assay reliably detected down to ≥ 10 copies of the synthetic *Sh Dra1* DNA standard when containing all four concentrations of betaine tested (Table 2).

When tested on *S. haematobium* gDNA, the Sh-RPA reliably detected down to 0.1 pg of gDNA in the presence of all four concentrations of betaine. However, in reactions containing lower concentrations of gDNA (0.01 pg and 1 fg), different outcomes were seen depending on betaine concentration, with inconsistent results (see Table 2).

No false-positive reactions (i.e. increase of ≥ 500 RFU from baseline fluorescence) occurred with the addition of each concentration of betaine, and reactions containing 12.5 M of betaine showed reduced background fluorescence compared to other concentrations.

### Effects of template DNA volume and betaine on Sh-RPA performance

Sh-RPA reactions containing 1, 6, 10.7, or 12.7 µl of gDNA extracted from a single egg with the addition of 0 M, 1 M, 2.5 M, or 12.5 M of betaine all gave robust positive amplification with no observed difference in analytical sensitivity (Table 2). However, reactions containing only 1 µl of gDNA showed a slower onset of amplification than reactions containing higher volumes of gDNA. Reactions containing 12.5 M of betaine were positive, but showed decreased overall fluorescence compared to reactions containing other concentrations of betaine. No assay inhibition was observed when all the water within the Sh-RPA reaction was replaced with the sample, simplifying reaction set-up.

### DNA isolated from urine samples

For urine samples U3, U16, and U17 (containing 9, 0.5, and 1.5 eggs per 10 ml of urine, respectively), all reactions gave robust positive results, regardless of the sample volume and concentration of betaine used (Table 2). Unfortunately, insufficient sample volumes for U19 and U20 (mean egg counts of 6 and 0.5 eggs per 10 ml of urine, respectively) were available to undertake all permutations of the assay. Given that the Sh-RPA has previously detected *S. haematobium* DNA in these samples without the addition of betaine [42], the decision was taken to omit the betaine-free reactions for U19 (both 6 µl and maximum sample volume) and U20 (maximum sample volume only) based on volume of sample remaining after undertaking the betaine-containing reactions (Table 2). Samples U19 and U20 both showed low-level amplification, and when the RFU cut-off was applied, several of the reactions were recorded as negative (Table 2). However, the observed fluorescent signal suggests that low-level amplification was taking place, although this may not be reliable.

Sample U19 demonstrated inconsistently positive results depending on the Sh-RPA conditions (Table 2). The amplification curves for sample U18 (mean egg count of 1 egg per 10 ml of urine) showed unusual levels of background fluorescence, as observed in previous studies that have tested this sample [29, 42]. When the RFU cut-off was applied, reactions containing 0 M, 1 M, and 2.5 M of betaine were positive with both 6 µl and maximum sample volume. False positives occurred sporadically in betaine-free negative control samples (Table 2).

In summary, reactions containing 2.5 M of betaine and the maximum sample volume of 12.7 µl yielded 100% sensitivity and specificity with clinical samples (Table 2). For all reactions, increasing the sample volume showed no negative effect on the performance of the Sh-RPA assay.

### Effects of different DNA extraction methods on Sh-RPA performance

The qPCR data from the different DNA extraction methods tested demonstrated that gDNA was obtained from all extracted samples, with variable mean cycle threshold (Ct) values for each kit (see Table 3).

**Fig. 1:**
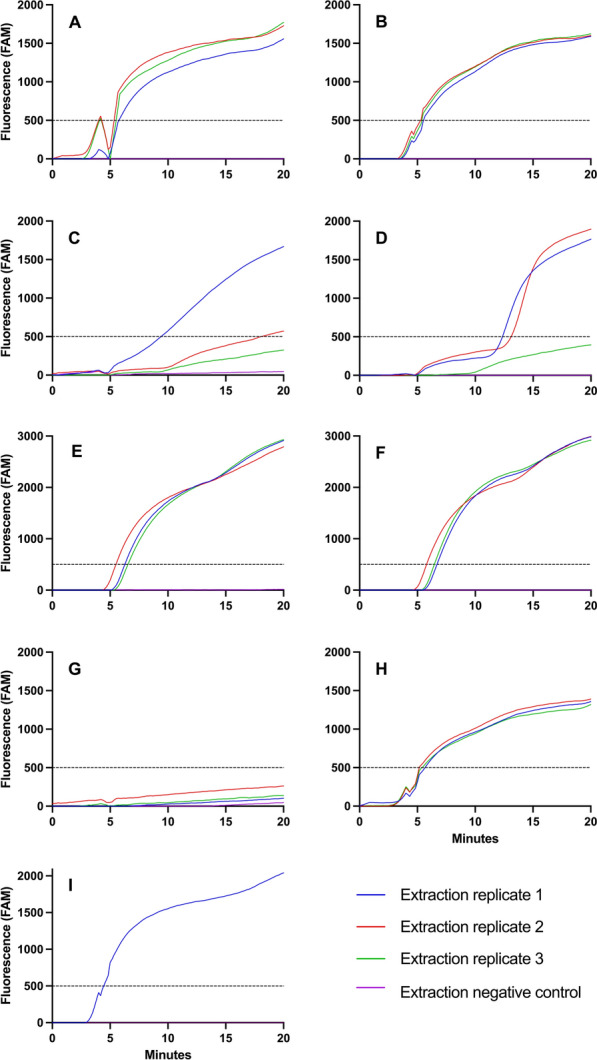
Comparison of Sh-RPA performance using DNA extracted from single *S. haematobium* eggs using different extraction methods. **A** Extracta DNA Prep (Quantabio) with 95 °C incubation. **B** Extracta DNA Prep (Quantabio) with room-temperature incubation. **C** DNeasy Blood & Tissue Kit (Qiagen) with 56 °C incubation. **D** DNeasy Blood & Tissue Kit (Qiagen) with room-temperature incubation. **E** SwiftX DNA Kit (Xpedite Diagnostics) with 95 °C incubation and modified protocol (see text). **F** SwiftX DNA Kit (Xpedite Diagnostics) with room-temperature incubation and modified protocol (see text). **G** Genesig Easy DNA/RNA Kit (PrimerDesign) with 95 °C incubation. **H** Direct egg addition to the Sh-RPA reaction with no extraction performed. **I** SpeedXtract Nucleic Acid Kit (Qiagen) with 95 °C incubation (single replicate extraction only, due to kit being discontinued). Dashed line represents threshold for a result to be considered positive (≥ 500 RFU)

Figure 1 shows the Sh-RPA amplification curves for *S. haematobium* DNA that had been extracted from single eggs using the different extraction methods (Table 3). Samples extracted using the Extracta DNA Prep (Quantabio) kit gave positive results with both the recommended sample lysis temperature of 95 °C and room temperature (±19 °C) (Fig. 1A, B). When using the DNeasy Blood & Tissue Kit (Qiagen), with both the lysis step temperatures tested (56 °C and RT ± 19 °C), reactions were not robust and showed unusually distorted amplification curves (Fig. 1C, D). Samples extracted using the modified SwiftX DNA Kit (Xpedite Diagnostics) extraction protocol, performed at both 95 °C and room temperature, gave positive results (Fig. 1E, F). However, all Sh-RPA reactions failed for samples extracted using the Genesig Easy DNA/RNA Kit (Primerdesign) kit (Fig. 1G).

Interestingly, when adding single eggs directly to the Sh-RPA reaction, positive amplification curves were achieved (Fig. 1H). The SpeedXtract Nucleic Acid Kit (Qiagen) gave positive results with rapid amplification of single egg DNA (Fig. 1I). All negative extraction controls gave negative Sh-RPA results.

## Discussion

Here, the Sh-RPA assay developed for the molecular diagnosis of *S. haematobium* infection [29, 30] was used in two different formulations: with and without the addition of betaine. Previous studies have demonstrated the improved specificity of molecular assays with the addition of betaine [34]. However, the addition of betaine has also been shown to reduce assay sensitivity as increasing amounts are incorporated into the assays. The primary objective for the inclusion of betaine in this study was to improve specificity by preventing false-positive reactions that are thought to occur due to secondary structure formation of the RPA primers and probe. This study additionally explored the effect of different concentrations of betaine on the sensitivity of the Sh-RPA assay.

Previous work with the Sh-RPA demonstrated an analytical LOD of 1 fg of gDNA [42]. Adding greater concentrations of betaine (2.5 M and 12.5 M) reduced sensitivity and resulted in an analytical LOD of 0.1 pg, but with occasional positives at concentrations of 0.01 pg and 1 fg. However, the clinical relevance of such a decrease in sensitivity appears to be of limited concern given the positive Sh-RPA results using clinical samples in this study, particularly when considering the benefits of improved specificity. For Sh-RPA reactions with extracts from clinical urine samples, the addition of 2.5 M of betaine did not reduce assay sensitivity. However, reduced sensitivity was observed with 12.5 M of betaine. This is consistent with previous studies showing that excessive betaine in isothermal molecular assays can be disadvantageous, slowing down the reaction and decreasing the final yield [46, 47].

With a view to potentially further increasing Sh-RPA sensitivity, our data also show that all of the water in the Sh-RPA reactions can be replaced by sample DNA extract, with no detrimental effect on the reaction. This highlights the tolerance of the Sh-RPA to sample inhibitors, whilst also simplifying reaction set-up. In combination, the addition of 2.5 M betaine, together with the maximum sample extraction volume (replacing all water), provided optimal assay sensitivity and specificity.

As part of this study, different simple and portable extraction methods were tested to assess their performance and compatibility with Sh-RPA. The Extracta DNA Prep kit (Quantabio) and the SwiftX DNA kit (Xpedite Diagnostics) showed highly promising results in terms of accuracy and usability, as well as having a much lower cost than the other kits tested. Interestingly, a very commonly used commercial and filter-column-based DNA extraction kit (DNeasy Blood & Tissue Kit, Qiagen) appeared to affect the performance of the RPA reaction in our study, with abnormal amplification curves observed when using this protocol. However, assay sensitivity was not affected. Furthermore, the Genesig Easy DNA/RNA Kit (Primerdesign), which has highly desirable characteristics for use in low-resource settings such as simplicity and lack of a heating step, gave negative results in our study. This is likely because the reagents in the kit had a detrimental effect on the recombinase enzymes, since the qPCR results confirmed the presence of DNA in the extract. Such variability in results depending on extraction kit/assay combination has been seen with other molecular diagnostics, such as LAMP, where different DNA extractions methods and protocols caused variations in sensitivity [48–51]. Of note, in our study, the use of heating steps as part of the sample lysis step was deemed unnecessary, with unheated replicates for each kit producing broadly similar Sh-RPA results. This makes the use of Sh-RPA in a point-of-care setting much more feasible, since no power source for heated incubation (e.g. heat blocks) would be needed, although this would have to be explored on a larger set of clinical samples.

These results confirm the need to give considerable attention to the sample preparation/DNA extraction stage when developing molecular diagnostic assays, as this is often the limiting factor for point-of-care implementation. As this study demonstrates, the assumption that all commercially available kits will be compatible with the downstream molecular assays being used for diagnosis may not be correct, and so robust testing is needed to ensure compatibility and optimal performance.

The primary limitation of this study is that only a small number of clinical samples were tested. This study serves as preliminary data and hypothesis testing, but larger-scale assessments of Sh-RPA in endemic settings should be carried out. Another limitation is that each sample was extracted in Zanzibar by removing an aliquot of the stored parent sample, and this aliquot may or may not have contained *S. haematobium* eggs. This limits the conclusions which can be drawn about performance with these clinical samples compared to egg microscopy measures, as egg presence/absence in the extraction will play a role in the sensitivity of Sh-RPA. This is particularly relevant when egg counts are very low. Again, larger studies testing more clinical samples would enable more advanced sensitivity testing. Urinary excretion of parasite cell-free DNA (cfDNA) may also contribute to assay sensitivity, and further work is needed to characterise whether egg or cfDNA presence affects assay results more significantly. Lastly, in this study these commercial extraction methods were only performed on eggs suspended in water, and further work would be required to compare their performance on eggs suspended in urine (e.g. spiked samples) to more accurately reflect clinical use.

If Sh-RPA is to be used as a diagnostic for a urogenital schistosomiasis elimination programme, a cost-effectiveness analysis would also need to be undertaken. The first step of this would be a direct comparison of the optimised Sh-RPA protocol with other diagnostics for urogenital schistosomiasis in an endemic setting. A major limiting factor on widespread use of Sh-RPA currently is the cost and availability of commercial RPA kits. Until these are more readily available at low cost, one cost-saving measure which could be employed is utilising half the recommended reaction components; i.e. processing each reaction in a total volume of 25 µl, which has been shown to yield good results in other work [52, 53]. Assessing the impact of lower sample volumes on Sh-RPA reactions would clarify whether this approach is feasible, and could affect any analysis of cost-effectiveness.

## Conclusions

In summary, this study shows that the addition of 2.5 M of betaine increases Sh-RPA specificity whilst having no observable negative impact on assay sensitivity. In addition, we also demonstrate that choice of DNA extraction method can have a significant impact on assay performance. This advances the assay towards fulfilling WHO’s schistosomiasis diagnostic target product profile [16], and therefore moves the assay further towards point-of-care use in endemic settings.

### Supplementary Information

Supplementary Material 1.

## Data Availability

All data generated or analysed during this study are included in this published article and its supplementary information files.

## References

[1] Colley DG, Bustinduy AL, Secor WE, King CH. Human schistosomiasis. Lancet. 2014;383:2253–64. 10.1016/S0140-6736(13)61949-2. 24698483 10.1016/S0140-6736(13)61949-2 PMC4672382

[2] Vos T, Lim SS, Abbafati C, Abbas KM, Abbasi M, Abbasifard M, et al. Global burden of 369 diseases and injuries in 204 countries and territories, 1990–2019: a systematic analysis for the global burden of disease study 2019. The Lancet. 2020;396:1204–22. 10.1016/S0140-6736(20)30925-9. 10.1016/S0140-6736(20)30925-9 PMC7567026 33069326

[3] GBD Compare | IHME Viz Hub n.d. http://vizhub.healthdata.org/gbd-compare. Accessed on 7 August 2021.

[4] Nemhauser JB, editor. CDC Yellow Book 2024: health information for international travel. Oxford: Oxford University Press; 2023.

[5] World Health Organization. Schistosomiasis and soil-transmitted helminthiases: number of people treated in 2018. Wkly Epidemiol Rec. 2019;94:601–12.

[6] McManus DP, Dunne DW, Sacko M, Utzinger J, Vennervald BJ, Zhou X-N. Schistosomiasis. Nat Rev Dis Primer. 2018;4:13. 10.1038/s41572-018-0013-8. 10.1038/s41572-018-0013-8 30093684

[7] International Agency for Research on Cancer (IARC). Schistosomes, liver flukes and helicobacter pylori, vol. 61. Lyon: IARC; 1994. PMC7681621 7715068

[8] Leutscher PDC, Ramarokoto C-E, Hoffmann S, Jensen JS, Ramaniraka V, Randrianasolo B, et al. Coexistence of urogenital schistosomiasis and sexually transmitted infection in women and men living in an area where *Schistosoma haematobium* is endemic. Clin Infect Dis. 2008;47:775–82. 10.1086/591127. 18680415 10.1086/591127

[9] Leutscher PDC, Høst E, Reimert CM. Semen quality in *Schistosoma haematobium* infected men in Madagascar. Acta Trop. 2009;109:41–4. 10.1016/j.actatropica.2008.09.010. 18950598 10.1016/j.actatropica.2008.09.010

[10] Patel P, Rose CE, Kjetland EF, Downs JA, Mbabazi PS, Sabin K, et al. Association of schistosomiasis and HIV infections: a systematic review and meta-analysis. Int J Infect Dis. 2021;102:544–53. 10.1016/j.ijid.2020.10.088. 33157296 10.1016/j.ijid.2020.10.088 PMC8883428

[11] Kjetland EF, Ndhlovu PD, Gomo E, Mduluza T, Midzi N, Gwanzura L, et al. Association between genital schistosomiasis and HIV in rural Zimbabwean women. AIDS. 2006;20:593–600. 10.1097/01.aids.0000210614.45212.0a. 16470124 10.1097/01.aids.0000210614.45212.0a

[12] Kjetland EF, Leutscher PDC, Ndhlovu PD. A review of female genital schistosomiasis. Trends Parasitol. 2012;28:58–65. 10.1016/j.pt.2011.10.008. 22245065 10.1016/j.pt.2011.10.008

[13] Kayuni S, Lampiao F, Makaula P, Juziwelo L, Lacourse EJ, Reinhard-Rupp J, et al. A systematic review with epidemiological update of male genital schistosomiasis (MGS): a call for integrated case management across the health system in sub-Saharan Africa. Parasite Epidemiol Control. 2019;4:e00077. 10.1016/j.parepi.2018.e00077. 30662962 10.1016/j.parepi.2018.e00077 PMC6324017

[14] King CH. Toward the elimination of schistosomiasis. N Engl J Med. 2009;360:106–9. 10.1056/NEJMp0808041. 19129524 10.1056/NEJMp0808041

[15] Rollinson D, Knopp S, Levitz S, Stothard JR, Tchuem Tchuenté L-A, Garba A, et al. Time to set the agenda for schistosomiasis elimination. Acta Trop. 2013;128:423–40. 10.1016/j.actatropica.2012.04.013. 22580511 10.1016/j.actatropica.2012.04.013

[16] World Health Organization. Ending the neglect to attain the Sustainable Development Goals: A road map for neglected tropical diseases 2021–2030 2020.

[17] Gass K. Time for a diagnostic sea-change: rethinking neglected tropical disease diagnostics to achieve elimination. PLoS Negl Trop Dis. 2020;14:e0008933. 10.1371/journal.pntd.0008933. 33382694 10.1371/journal.pntd.0008933 PMC7774841

[18] Land KJ, Boeras DI, Chen X-S, Ramsay AR, Peeling RW. REASSURED diagnostics to inform disease control strategies, strengthen health systems and improve patient outcomes. Nat Microbiol. 2019;4:46–54. 10.1038/s41564-018-0295-3. 30546093 10.1038/s41564-018-0295-3 PMC7097043

[19] Le L, Hsieh MH. Diagnosing urogenital schistosomiasis: dealing with diminishing returns. Trends Parasitol. 2017;33:378–87. 10.1016/j.pt.2016.12.009. 28094201 10.1016/j.pt.2016.12.009

[20] de Vlas SJ, Gryseels B. Underestimation of *Schistosoma mansoni* prevalences. Parasitol Today. 1992;8:274–7. 10.1016/0169-4758(92)90144-Q. 15463638 10.1016/0169-4758(92)90144-Q

[21] Wilson A, van Dam GJ, Kariuki TM, Farah IO, Deelder AM, Coulson PS. The detection limits for estimates of infection intensity in schistosomiasis mansoni established by a study in non-human primates. Int J Parasitol. 2006;36:1241–4. 10.1016/j.ijpara.2006.07.002. 16930605 10.1016/j.ijpara.2006.07.002

[22] Savioli L, Hatz C, Dixon H, Kisumku UM, Mott KE. Control of morbidity due to *Schistosoma haematobium* on Pemba Island: egg excretion and hematuria as indicators of infection. Am J Trop Med Hyg. 1990;43:289–95. 10.4269/ajtmh.1990.43.289. 2121056 10.4269/ajtmh.1990.43.289

[23] King CH, Bertsch D. Meta-analysis of urine heme dipstick diagnosis of *Schistosoma haematobium* infection, including low-prevalence and previously-treated populations. PLoS Negl Trop Dis. 2013;7:e2431. 10.1371/journal.pntd.0002431. 24069486 10.1371/journal.pntd.0002431 PMC3772022

[24] Stothard JR, Sousa-Figueiredo JC, Standley C, Van Dam GJ, Knopp S, Utzinger J, et al. An evaluation of urine-CCA strip test and fingerprick blood SEA-ELISA for detection of urinary schistosomiasis in schoolchildren in Zanzibar. Acta Trop. 2009;111:64–70. 10.1016/j.actatropica.2009.02.009. 19426665 10.1016/j.actatropica.2009.02.009

[25] Doenhoff MJ, Chiodini PL, Hamilton JV. Specific and sensitive diagnosis of schistosome infection: can it be done with antibodies? Trends Parasitol. 2004;20:35–9. 10.1016/j.pt.2003.10.019. 14700588 10.1016/j.pt.2003.10.019

[26] Corstjens PLAM, de Dood CJ, Knopp S, Clements MN, Ortu G, Umulisa I, et al. Circulating anodic antigen (CAA): a highly sensitive diagnostic biomarker to detect active *Schistosoma* infections—improvement and use during SCORE. Am J Trop Med Hyg. 2020;103:50–7. 10.4269/ajtmh.19-0819. 32400344 10.4269/ajtmh.19-0819 PMC7351307

[27] Guegan H, Fillaux J, Charpentier E, Robert-Gangneux F, Chauvin P, Guemas E, et al. Real-time PCR for diagnosis of imported schistosomiasis. PLoS Negl Trop Dis. 2019;13:e0007711. 10.1371/journal.pntd.0007711. 31509538 10.1371/journal.pntd.0007711 PMC6756557

[28] Keller D, Rothen J, Dangy J-P, Saner C, Daubenberger C, Allan F, et al. Performance of a real-time PCR approach for diagnosing *Schistosoma haematobium* infections of different intensity in urine samples from Zanzibar. Infect Dis Poverty. 2020;9:128. 10.1186/s40249-020-00726-y. 32887642 10.1186/s40249-020-00726-y PMC7487541

[29] Archer J, Barksby R, Pennance T, Rostron P, Bakar F, Knopp S, et al. Analytical and clinical assessment of a portable, isothermal recombinase polymerase amplification (RPA) assay for the molecular diagnosis of urogenital schistosomiasis. Molecules. 2020;25:4175. 10.3390/molecules25184175. 32933094 10.3390/molecules25184175 PMC7570534

[30] Archer J, Patwary FK, Sturt AS, Webb EL, Phiri CR, Mweene T, et al. Validation of the isothermal *Schistosoma haematobium* recombinase polymerase amplification (RPA) assay, coupled with simplified sample preparation, for diagnosing female genital schistosomiasis using cervicovaginal lavage and vaginal self-swab samples. PLoS Negl Trop Dis. 2022;16:e0010276. 10.1371/journal.pntd.0010276. 35286336 10.1371/journal.pntd.0010276 PMC8947142

[31] Aebischer A, Wernike K, Hoffmann B, Beer M. Rapid genome detection of Schmallenberg virus and bovine viral diarrhea virus by use of isothermal amplification methods and high-speed real-time reverse transcriptase PCR. J Clin Microbiol. 2014;52:1883–92. 10.1128/JCM.00167-14. 24648561 10.1128/JCM.00167-14 PMC4042763

[32] Rees WA, Yager TD, Korte J, Von Hippel PH. Betaine can eliminate the base pair composition dependence of DNA melting. Biochemistry. 1993;32:137–44. 10.1021/bi00052a019. 8418834 10.1021/bi00052a019

[33] Zou Y, Mason MG, Botella JR. Evaluation and improvement of isothermal amplification methods for point-of-need plant disease diagnostics. PLoS ONE. 2020;15:e0235216. 10.1371/journal.pone.0235216. 32598374 10.1371/journal.pone.0235216 PMC7323990

[34] Luo G-C, Yi T-T, Jiang B, Guo X, Zhang G-Y. Betaine-assisted recombinase polymerase assay with enhanced specificity. Anal Biochem. 2019;575:36–9. 10.1016/j.ab.2019.03.018. 30930198 10.1016/j.ab.2019.03.018

[35] Henke W, Herdel K, Jung K, Schnorr D, Loening SA. Betaine improves the PCR amplification of GC-rich DNA sequences. Nucleic Acids Res. 1997;25:3957–8. 10.1093/nar/25.19.3957. 9380524 10.1093/nar/25.19.3957 PMC146979

[36] Özay B, McCalla SE. A review of reaction enhancement strategies for isothermal nucleic acid amplification reactions. Sens Actuators Rep. 2021;3:100033. 10.1016/j.snr.2021.100033. 10.1016/j.snr.2021.100033

[37] Kersting S, Rausch V, Bier FF, von Nickisch-Rosenegk M. Multiplex isothermal solid-phase recombinase polymerase amplification for the specific and fast DNA-based detection of three bacterial pathogens. Microchim Acta. 2014;181:1715–23. 10.1007/s00604-014-1198-5. 10.1007/s00604-014-1198-5 PMC4167443 25253912

[38] Lobato IM, O’Sullivan CK. Recombinase polymerase amplification: basics, applications and recent advances. Trends Anal Chem TRAC. 2018;98:19–35. 10.1016/j.trac.2017.10.015. 10.1016/j.trac.2017.10.015 PMC7112910 32287544

[39] Lewis FA, Liang Y, Raghavan N, Knight M. The NIH-NIAID Schistosomiasis Resource Center. PLoS Negl Trop Dis. 2008;2:e267. 10.1371/journal.pntd.0000267. 18665228 10.1371/journal.pntd.0000267 PMC2480520

[40] Cody JJ, Ittiprasert W, Miller AN, Henein L, Mentink-Kane MM, Hsieh MH. The NIH-NIAID Schistosomiasis Resource Center at the biomedical research institute: molecular redux. PLoS Negl Trop Dis. 2016;10:e0005022. 10.1371/journal.pntd.0005022. 27764112 10.1371/journal.pntd.0005022 PMC5072641

[41] Emery AM, Allan FE, Rabone ME, Rollinson D. Schistosomiasis collection at NHM (SCAN). Parasit Vectors. 2012;5:185. 10.1186/1756-3305-5-185. 22943137 10.1186/1756-3305-5-185 PMC3453491

[42] Rostron P, Pennance T, Bakar F, Rollinson D, Knopp S, Allan F, et al. Development of a recombinase polymerase amplification (RPA) fluorescence assay for the detection of *Schistosoma haematobium*. Parasit Vectors. 2019;12:514. 10.1186/s13071-019-3755-6. 31685024 10.1186/s13071-019-3755-6 PMC6827214

[43] Knopp S, Mohammed KA, Ali SM, Khamis IS, Ame SM, Albonico M, et al. Study and implementation of urogenital schistosomiasis elimination in Zanzibar (Unguja and Pemba islands) using an integrated multidisciplinary approach. BMC Public Health. 2012;12:1–13. 10.1186/1471-2458-12-930. 23110494 10.1186/1471-2458-12-930 PMC3533998

[44] Obeng BB, Aryeetey YA, de Dood CJ, Amoah AS, Larbi IA, Deelder AM, et al. Application of a circulating-cathodic-antigen (CCA) strip test and real-time PCR, in comparison with microscopy, for the detection of *Schistosoma haematobium* in urine samples from Ghana. Ann Trop Med Parasitol. 2008;102:625–33. 10.1179/136485908X337490. 18817603 10.1179/136485908X337490

[45] Melchers NVSV, van Dam GJ, Shaproski D, Kahama AI, Brienen EAT, Vennervald BJ, et al. Diagnostic performance of *Schistosoma* real-time PCR in urine samples from Kenyan children infected with *Schistosoma haematobium*: day-to-day variation and follow-up after praziquantel treatment. PLoS Negl Trop Dis. 2014;8:e2807. 10.1371/journal.pntd.0002807. 24743389 10.1371/journal.pntd.0002807 PMC3990496

[46] Ma C, Wang Y, Zhang P, Shi C. Accelerated isothermal nucleic acid amplification in betaine-free reaction. Anal Biochem. 2017;530:1–4. 10.1016/j.ab.2017.04.017. 28457896 10.1016/j.ab.2017.04.017

[47] Mok E, Wee E, Wang Y, Trau M. Comprehensive evaluation of molecular enhancers of the isothermal exponential amplification reaction. Sci Rep. 2016;6:37837. 10.1038/srep37837. 27910874 10.1038/srep37837 PMC5133538

[48] Ablordey A, Ahotor E, Narh CA, King SA, Cruz I, Ndung’u JM, et al. Evaluation of different DNA extraction methods and loop-mediated isothermal amplification primers for the detection of *Mycobacterium ulcerans* in clinical specimens. BMC Infect Dis. 2021;21:598. 10.1186/s12879-021-06308-z. 34162342 10.1186/s12879-021-06308-z PMC8220662

[49] Price M, Cyrs A, Sikasunge CS, Mwansa J, Lodh N. Testing the infection prevalence of *Schistosoma mansoni* after mass drug administration by comparing sensitivity and specificity of species-specific repeat fragment amplification by PCR and loop-mediated isothermal amplification. Am J Trop Med Hyg. 2019;101:78–83. 10.4269/ajtmh.19-0121. 31115299 10.4269/ajtmh.19-0121 PMC6609171

[50] Shuryaeva AK, Malova TV, Tolokonceva AA, Karceka SA, Gordukova MA, Davydova EE, et al. Development and application of LAMP assays for the detection of enteric adenoviruses in feces. Microbiol Spectr. 2022;10:e00516-e522. 10.1128/spectrum.00516-22. 35862966 10.1128/spectrum.00516-22 PMC9430467

[51] Sun Y, Zhao L, Zhao M, Zhu R, Deng J, Wang F, et al. Four DNA extraction methods used in loop-mediated isothermal amplification for rapid adenovirus detection. J Virol Methods. 2014;204:49–52. 10.1016/j.jviromet.2014.04.006. 24747588 10.1016/j.jviromet.2014.04.006

[52] Lillis L, Siverson J, Lee A, Cantera J, Parker M, Piepenburg O, et al. Factors influencing recombinase polymerase amplification (RPA) assay outcomes at point of care. Mol Cell Probes. 2016;30:74–8. 10.1016/j.mcp.2016.01.009. 26854117 10.1016/j.mcp.2016.01.009 PMC4818709

[53] Mesquita SG, Lugli EB, Matera G, Fonseca CT, Caldeira RL, Webster B. Development of real-time and lateral flow recombinase polymerase amplification assays for rapid detection of *Schistosoma mansoni*. Front Microbiol. 2022. 10.3389/fmicb.2022.1043596. 36466644 10.3389/fmicb.2022.1043596 PMC9716991

