# Supplementary material for: Refining the Schistosoma haematobium recombinase polymerase amplification (Sh-RPA) assay: moving towards point-of-care use in endemic settings
Source: Parasit Vectors. 2024 Jul 28;17:321. doi: 10.1186/s13071-024-06380-9 (PMC11283713; doi:10.1186/s13071-024-06380-9)
Supplement: Supplementary file 1 — Supplementary Material 1. [file 13071_2024_6380_MOESM1_ESM.docx]

SUPPLEMENTARY

**Supplementary Table S1**: Nucleic acid sequences used in this study. 6FAM = 6-carboxyfluoroscein fluorophore; THF = Tetrahydrofuran; BHQ1 = Black Hole Quencher.

| **Use in study** | **Nucleic acid sequence** |
| --- | --- |
| Synthetic DNA standard incorporating Dra1 sequence (bold) | CCTTGGTCACGTGATTTTCAGTTTGCCCCACCCTGATGCTGGCTG  CCCCACCTCGACCGGCATAAGGTGGAGC**GATCTCACCTATCAG**  **ACGAAACAAAGAAAATTTTAAAATTGTTGGTGGAAGTGCCTG**  **TTTCGAATATCTCCGGAATGGTTGGTCGTATCGTTGTGAAAAT**  **TGTTTCATATTATTGGTGAC** |
| Forward Sh-RPA primer | 5’ATCTCACCTATCAGACGAAACAAAGAAAAT 3’ |
| Reverse Sh-RPA primer | 5’AATATGAAACAATTTTCACAACGATACGAC 3’ |
| Fluorescent Sh-RPA probe | 5’AATTGTTGGTGGAAGTGCCTGTTTCGCAA(6FAM)(THF)(BHQ1)CTCCGGAATGGTTG(c3-Spacer) 3’ |

**Supplementary Figure 1:** Sh-RPA amplification curves for the synthetic *Sh* Dra1 DNA standards containing **(A)** 5x10^8^, 5x10^2^ or 5x10^1^ copies with and without 2.5M of betaine. The dip in the amplification curve for the reactions containing 5x10^8^ copies occurs due to the manual mix of the reactions at four minutes from the start of the reaction. **(B)** 5x10^1^ and 1x10^1^ copies of the synthetic *Sh* Dra1 DNA standard with 1M, 2.5M, or 12.5M of betaine. Dashed line represents threshold for a result to be considered positive (≥ 500 RFU).
